# Patients with pelvic fractures due to falls: A paradigm that contributed to autopsy-based audit of trauma in Greece

**DOI:** 10.1186/1752-2897-5-2

**Published:** 2011-01-08

**Authors:** Iordanis N Papadopoulos, Nikolaos K Kanakaris, Stefanos Bonovas, George Konstantoudakis, Konstantina Petropoulou, Spyridon Christodoulou, Olympia Kotsilianou, Christos Leukidis

**Affiliations:** 1 National & Kapodistrian University of Athens, Attikon University General Hospital, Fourth Surgery Department, 1 Rimini Street, 124 62, Athens, Greece; 2 Department of Epidemiological Surveillance & Intervention, Center for Diseases Control & Prevention, Athens, Greece; 3 The Athens Forensic Medical Department, Ministry of Justice, 10 Anapaphseos Street, 116 36, Athens, Greece

## Abstract

**Background:**

Evaluation of the pelvic fractures (PFx) population in auditing effective components of trauma care is the subject of this study.

**Methods:**

A retrospective, case-control, autopsy-based study compared a population with PFx to a control-group using a *template *with trauma outcome variables, which included demographics, ICD-9, intention, mechanisms, toxicology, Abbreviated Injury Scale (AIS-90), Injury Severity Score (ISS), causes of haemorrhage, comorbidity, survival time, pre-hospital response, in hospital data, location of death, and preventable deaths.

**Results:**

Of 970 consecutive patients with fatal falls, 209 (21.5%) had PFx and constituted the PFx-group while 761 (78.5%) formed the control-group.

Multivariate analysis showed that gender, age, intention, and height of fall were risk factors for PFx. A 300% higher odds of a psychiatric history was found in the PFx-group compared to the control-group (p < 0.001).

The median ISS was 50 (17-75) for the PFx-group and 26 (1-75) for the control-group (p < 0.0001). There were no patients with an ISS less than 16 in the PFx group.

Associated injuries were significantly more common in the PFx-group than in the control-group. Potentially preventable deaths (ISS < 75) constituted 78% (n = 163) of the PFx-group. The most common AIS3-5 injuries in the potentially preventable subset of patients were the lower extremities in 133 (81.6%), thorax in 130 (79.7%), abdomen/pelvic contents in 99 (60.7%), head in 95 (58.3%) and the spine in 26 (15.9%) patients.

A subset of 126 (60.3%) potentially preventable deaths in the PFx-group had at least one AIS-90 code other than the PFx, denoting major haemorrhage. Deaths directly attributed to PFx were limited to 6 (2.9%).

The median survival time was 30 minutes for the PFx-group and 20 hours for the control-group (p < 0.001). For a one-group increment in the ISS-groups, the survival rates over the post-traumatic time intervals were reduced by 57% (p < 0.0001).

Pre-hospital mortality was significantly higher in the PFx-group i.e. 70.3% of the PFx-group versus 42.7% of the control-group (*p *< 0.001).

**Conclusions:**

The PFx-group shared common causative risk factors, high severity and multiplicity of injuries that define the PFx-group as a paradigm of injury for audit. This reduced sample of autopsies substantially contributed to the audit of functional, infrastructural, management and prevention issues requiring transformation to reduce mortality.

## Background

An evidence based transformation of trauma care is nowadays necessary. Nationally based trauma databanks and prospective research have substantially contributed to this task. Autopsy based audit remains a useful option, but selecting the trauma population in order to audit trauma care is an essential issue.

Pelvic fractures (PFx) are recognized as severe injuries that leave 'a record' on the patients with respect to the direction and magnitude of the force of impact, which helps to predict the patterns of commonly associated organ injuries [1]. In a recent publication [2], PFx related fatalities were used as a basis to audit the trauma care system in Greece.

The rational and the theoretical basis of selecting PFx to audit trauma care is that the pelvis is an inherently stable structure, hence large energy absorption is required to produce a PFx. Energy dissipation causes many life threatening associated injuries. Hence, selecting the population with PFx should select a reproducible population, thus allowing data comparison.

The present study used PFx population due to falls as a paradigm of injury in order to define its contribution in auditing trauma care.

## Patients and Methods

### Objectives

The primary objective of this study was to define the role of the PFx population due to falls in auditing effective components of the trauma care delivery system.

### Design

This is a retrospective, case-control study based on autopsy findings.

### Settings

To achieve the defined objectives the course from injury to death of all consecutive casualties who suffered fatal falls and were subjected to a formal medico legal autopsy in the Athens Forensic Medical Department from January 1, 1996 to February 8, 2001, was reviewed and a set of trauma outcome variables (template) was constructed.

### The template with trauma outcome variables was used for comparisons of the PFx group versus the control group

Data to construct the template with outcome variables were extracted from the *Attica - Trauma Audit and Research Autopsy-Based Registry *and included the following: demographics; location of injury; intention for the injury; mechanism of injury classified according to the International Statistical Classification of diseases, injuries and causes of death - 9th revision (ICD-9) [3]; toxicology; distribution of all injuries in the anatomic regions and their severity codes as defined by the Abbreviated Injury Scale - 1990 revision (AIS-90) [4]; potential causes of major haemorrhage other than PFx; postmortem Injury Severity Score (ISS) [5]; posttraumatic survival time; co-morbidity data; location where death occurred; surgical procedures performed; complication rates; estimation of deaths directly attributed to PFx; as well as estimation of the proportion of potentially preventable deaths.

### Mechanisms of injury

With respect to mechanisms of injury, only patients with fatal falls were included in the study and were split into simple falls (SF) and non-simple falls (non-SF). SF were defined, according to ICD-9 codes E885, E886, E884.2, as a fall with the victim standing or sitting [6].

### Diagnosis and classification of injuries

The diagnoses of injuries were based on the autopsy examinations but hospital data were also incorporated when they were available. All recorded injuries were retrospectively included in the registry. Consequently injuries were classified according to AIS and ISS by a single coder (NK), and reviewed by the first author.

### Causes of major haemorrhage

In order to identify potential causes of major haemorrhage other than PFx, a list of coded injuries shown in Table 1 was selected from the AIS-90 and their presence or absence in the PFx group were evaluated [2].

**Table 1:** Injuries potentially causing major haemorrhage in trauma patients other than the pelvic fractures, as previously used [2].

Description of Injury	AIS-90 codes
Carotid artery	2.2.02.04.3, 3.2.02.**.*, 3.2.04.**.*
Jugular vein	3.2.06.**.*, 3.2.08.**.*
Penetrating injuries with blood loss >20% by volume	1.60.06.3, 2.1.60.06.3, 3.1.60.06.3, 5.1.60.06.3, 7.1.60.06.3, 8.1.60.06.3
Skin laceration with blood loss >20% by volume	1.1.06.06.3, 2.1.06.06.3, 3.1.06.06.3, 4.1.06.06.3, 5.1.06.06.3, 7.1.06.06.3, 8.1.06.06.3
Skin avulsion with blood loss >20% by volume	1.1.08.06.3, 2.1.08.06.3, 3.1.08.06.3, 4.1.08.06.3, 5.1.08.06.3, 7.1.08.06.3, 8.1.08.06.3
Aorta	4.2.02.**.*, 5.2.02.**.*
Innominate vessels	4.2.04.**.*, 4.2.06.**.*
Pulmonary vessels	4.2.10.**.*, 4.2.12.**.*
Subclavian vessels	4.2.14.**.*, 4.2.16.**.*
Vena cava	4.2.18.**.*, 5.2.12.**.*
Myocardium	4.4.12.**.*, 4.4.10.**.*, 4.4.13.**.*
Celiac artery	5.2.04.**.*
Named abdominal vessels	5.2.14.**.*, 5.2.16.**.*
Liver	5.4.18.14.3, 5.4.18.24.3
Spleen	5.4.42.**.*
Kidney	5.4.16.14.3, 5.4.16.24.3, 5.4.16.40.3
Mesentery with blood loss >20% by volume	5.4.20.24.3
Omentum laceration major with blood loss >20% by volume	5.4.22.24.3
Limb crush	7.1.30.**.*, 8.1.30.**.*
Traumatic amputation (except fingers, toes)	7.1.10.**.*, 8.1.10.**.*
Axillary vessels major laceration	7.2.02.08.3, 7.2.04.06.3
Brachial vessels major laceration	7.2.06.08.3, 7.2.08.06.3
Major lacerations of other named vessels of upper limb	7.2.10.08.3, 7.2.12.06.3
Femoral vessels	8.2.02.**.*, 8.2.04.**.*
Popliteal vessels	8.2.06.**.*, 8.2.08.**.*
Major lacerations of other named vessels of lower limb	8.2.10.**.*, 8.2.12.**.*

*Asterisks that follow the AIS-90 codes indicate that the complete range of injuries was included. AIS-90: Abbreviated Injury Scale - 1990 revision. Severity of injuries ranged from 1 to 6, with AIS1 being minor, AIS2 moderate, AIS3 serious, AIS4 severe, AIS5 critical, and AIS6 an unsurvivable injury.

### Deaths attributed to pelvic fractures

Deaths attributable to PFx were based on modification of the definition used by Demetriades and colleagues [7] and included patients with: 1) severity of PFx equal to or higher than AIS4; 2) absence of any other injury in any other anatomic region with severity equal to or higher than AIS4; 3) death should have occurred within the first 48 hours after injury in order to be attributable to PFx; and 4) the recorded injuries with an AIS-90 code shown in Table 1 which denote a potential cause of major haemorrhage other than those related to PFx.

### Potentially preventable deaths

Deaths were defined as potentially preventable/salvageable under optimal care when the patients had an ISS equal or less than 74 in the context that the injuries do not necessarily lead to death. Non-preventable/unsalvageable deaths were defined when the ISS was equal to 75.

### Statistical analysis

Nonparametric statistical tests, i.e. Mann-Whitney or Chi-squared tests as well as logistic regression models were used when appropriate. Multivariate analyses were adjusted for gender, age, intention for injury, and height of fall. The level of *p *< 0.05 was chosen to indicate statistical significance. All statistical tests were performed using Stata statistical software, version 6.0 (Stata Corp, College Station, TX, USA).

### Ethics

The ethics committees of the University General Hospital 'Attikon' and the Forensic Medical Department of Athens approved this study.

## Results

### Sample size

The number of deaths attributable to falls (ICD-9, E50) reported by the National Statistics Service of Greece [8] throughout Greece for the period 1996 to 2000 was 2,102. The present study included 970 (46%) fatalities due to falls for approximately the same period of time i.e. January 1, 1996 to February 8, 2001.

### Pelvic fracture group, control group and rates

The dataset consisted of 970 patients who suffered blunt trauma due to falls, of whom 209 (21.5%) had PFx and constituted the PFx group. The remaining 761 (78.5%) without PFx constituted the control group.

The PFx group represented 4.2% of the total 5,007 all-causes trauma fatalities excluding those that were due to drowning and poisoning, who were subjected to formal autopsy during the study period. PFx rates increased from 1996 to 2000 (Pearson chi-squared [4 df] = 15.20, *p *= 0.004).

### Risk factors for pelvic fractures

In order to identify risk factors for PFx, a multivariate logistic regression model was constructed. PFx was taken as the dependent variable and gender, age, intention for injury, and height of fall as the independent variables. The analysis revealed that all the above factors influenced the probability of subjects suffering a PFx after a fall. Consequently, all of the following analyses were adjusted for gender, age, intention, and height of fall.

### Gender

The PFx group included 125 (59.8%) males and 84 (40.2%) females, and the control group 501 (65.8%) males and 260 (34.2%) females. After adjustment for age, height of fall, and intention, it was shown that males had 28% lower odds of developing a PFx following a fall compared to females, but this was marginally statistically insignificant (OR = 0.723, 95% CI: 0.521-1.003, *p *= 0.052).

### Age

The PFx group had a median age of 55 (15-96) years and a mean age (± SD) of 54 (± 20) years, while the control group had a median of 66 (range 1-99) years and a mean (± SD) of 60.6 (± 21.5) years. The PFx group was statistically significantly younger than the controls (two-sample Wilcoxon rank-sum [Mann-Whitney] test, *p *< 0.0001). The test is a nonparametric one that is suitable for comparison, since the distribution of age was not normal (Shapiro-Wilk W test for normal distribution of variable "age", *p *< 0.0001).

When age was used as a continuous variable and examined as a risk factor for PFx, the multivariate logistic regression analysis (adjusted for gender, height of fall, and intention) showed that age was a notable predictor. For a one-year difference in age throughout the age range of patients, the odds ratio was equal to 0.988 (95% CI: 0.981-0.996, *p *= 0.002). This finding means that older people appear to have a significantly lower probability of suffering a PFx after a fall compared to younger subjects.

### Location of injury

Of the 209 patients of the PFx group, 163 (78%) were injured in the Greater Athens region, 10 (4.8%) in the provinces, and 36 (17.2%) at another or unknown location.

Of the 761 patients of the control group, 500 (65.7%) were injured in the Greater Athens region, 70 (9.2%) in the provinces, and the remaining 191 (25.1%) at another or unknown location.

Comparison of the above percentages revealed a substantial difference (Pearson chi-squared [2 df] = 11.29, *p *= 0.004) and indicated that a fall occurring in the Greater Athens region had a greater probability of causing a PFx than a fall occurring in the provinces.

### Intention for the injury

Stratification of the intention of the injury revealed that for the PFx group suicidal falls were the most common subset with 114 (54.5%), followed by unintentional falls with 54 (25.8%), while those for whom the cause could not be determined was 39 (18.7%), and falls due to assaults 2 (1%).

The control group comprised 532 (69.9%) subjects of unintentional falls, 143 (18.8%) of suicidal falls, 79 (10.4%) for whom the cause could not be determined and 7 (0.9%) due to assaults. The intention for the injury was associated with the probability of having a PFx (Pearson chi-squared [2 df] = 138.17, *p *< 0.001). In particular, those who had intended to commit suicide had a higher probability of developing a PFx after a fall compared to those who did not have such an intention, (see Additional file 1).

### Mechanism of injury: simple falls versus non-simple falls

The mechanism of injury was split into simple falls and non-simple falls. The commonest mechanism of injury in the PFx group was non-SF in 155 (74.2%), followed by SF in 42 (20.1%). The height of fall was unknown in 12 (5.7%).

The commonest mechanism of injury in the control group was SF in 412 (54.1%), followed by non-SF in 317 (41.7%). The height of fall was unknown in 32 (4.2%). Comparison indicates that the mechanism of injury is associated with the probability of having a PFx (Pearson chi-squared [2 df] = 77.06, *p *< 0.001). In particular, non-SF appear to have a significantly higher probability of resulting in a PFx compared to SF, (see Additional file 2).

### Height of fall

The median height of fall of the non-SF was 13 (3-200) metres for the PFx group and 9 (1-80) metres for the control group. When the height of fall was used as a continuous variable and examined as a risk factor for a PFx, the multivariate logistic regression analysis (adjusted for gender, age, and intention) revealed that the height of fall was a notable predictor, because for a one-metre increment in the height of fall, the odds ratio was equal to 1.012 (95% CI: 1.006-1.019, *p *< 0.001).

### Toxicology

Toxicology screening was performed in 192 of 209 (91.9%) of the PFx group and in 462 of 761 (60.7%) of the control group. Alcohol or illegal drugs of any blood concentration were found in 33 (17.2%) of the PFx group and in 89 (19.3%) of the control group, but there was no significant difference between the groups (Pearson chi-squared [1 df] = 0.385, *p *= 0.54).

### AIS-90 anatomic regions and severity codes

All injuries observed in the 209 patients of the PFx group were classified according to anatomic regions and the AIS-90 severity codes are shown in Table 2. A high severity as well as multiplicity and complexity of injuries were documented.

**Table 2:** The 209 patients with pelvic fractures and their injuries categorised according to the Abbreviated Injury Scale-90 severity codes and body regions. This classification offers a comprehensive and reproducible overview of the encountered spectrum of severity and the multiplicity of injuries.

	Total	**ISS ≥ 16 to < 75**, 163 (78%) patients (potentially preventable deaths)				**ISS = 75**, 46 (22%) patients (nonpreventable deaths)	
**AIS-90** **anatomic regions**	**AIS1-6:** **2,369 injuries**, **in** **209 patients**. **N, (%)**	**AIS3: 461** **injuries**, **in** **153** **patients**, **N, (%)**	**AIS4:** **224 injuries, in** **131 patients**. **N, (%)**	**AIS5:** **190 injuries, in** **119 patients**. **N, (%)**	**AIS 3 + 4 + 5: 875** **injuries**, **in** **163** **patients**. **N, (%)**	**AIS1-5:** **554** **injuries**, **in** **46** **patients**. **N, (%)**	**AIS6:** **55** **injuries**, **in** **42** **patients**. **N, (%)**

**1. Head**							

Injuries	328 (13.8)	119 (25.8)	45 (20.1)	6 (3.2)	170 (19.4)	68 (12.3)	17 (30.9)

Patients	155 (74.2)	92 (60.1)	30 (22.9)	5 (4.2)	95 (58.3)	29 (63)	17 (40.5)

**2. Face**							

Injuries	231 (9.7)	2 (0.4)	2 (0.9)	0 (0)	4 (0.5)	53 (9.6)	0 (0)

Patients	110 (52.6)	2 (1.3)	2 (1.5)	0 (0)	4 (2.4)	26 (56.5)	0 (0)

**3. Neck**							

Injuries	16 (0.7)	0 (0)	0 (0)	1 (0.5)	1 (0.1)	2 (0.4)	0 (0)

Patients	14 (6.7)	0 (0)	0 (0)	1 (0.8)	1 (0.6)	2 (4.3)	0 (0)

**4. Thorax**							

Injuries	492 (20.8)	42 (9.1)	96 (42.9)	139 (73.2)	277 (31.7)	106 (19.1)	16 (29.1)

Patients	191 (91.4)	39 (25.5)	84 (64.1)	93 (78.1)	130 (79.7)	42 (91.3)	16 (38.1)

**5. Abdominal & pelvic contents**							

Injuries	313 (13.2)	81 (17.6)	66 (29.5)	3 (1.6)	150 (17.1)	70 (12.6)	8 (14.5)

Patients	153 (73.2)	71 (46.4)	63 (48.1)	3 (2.5)	99 (60.7)	35 (76.1)	7 (16.7)

**6. Spine**							

Injuries	103 (4.3)	15 (3.2)	2 (0.9)	12 (6.3)	29 (3.3)	25 (4.5)	14 (25.4)

Patients	68 (32.5)	13 (8.5)	2 (1.5)	11 (9.2)	26 (15.9)	15 (32.6)	11 (26.2)

**7. Upper extremities**							

Injuries	286 (12.1)	43 (9.3)	0 (0)	0 (0)	43 (4.9)	78 (14.1)	0 (0)

Patients	136 (65.1)	20 (13.1)	0 (0)	0 (0)	20 (12.3)	34 (73.9)	0 (0)

**8. Lower extremities** **(all injuries)**							

Injuries	570 (24.1)	159 (34.5)	13 (5.8)	28 (14.7)	200 (22.9)	145 (26.2)	0 (0)

Patients	209 (100)	105 (68.6)	13 (9.9)	28 (23.5)	133 (81.6)	46 (100)	0 (0)

**8. Lower extremities** (Pelvic fracture codes only) 8.5.26.**.*, 8.5.28.**.*, 8.5.30.**.*)							

Injuries	214 (9)	79 (17.1)	13 (5.8)	28 (14.7)	120 (13.7)	47 (8.5)	0 (0)

Patients	209 (100)	77 (50.3)	13 (9.9)	28 (23.5)	118 (72.4)	46 (100)	0 (0)

**9. External**							

Injuries	30 (1.3)	0 (0)	0 (0)	1 (0.5)	1 (0.1)	7 (1.3)	0 (0)

Patients	17 (8.1)	0 (0)	0 (0)	1 (0.8)	1 (0.6)	3 (6.5)	0 (0)

*The asterisks that follow the AIS-90 codes indicate that the complete range of injuries was included; AIS-90: Abbreviated Injury Scale, 1990 revision; ISS: Injury Severity Score.

Analysis of 163 (78% of the total 209) potentially preventable deaths with an ISS over 16 but less than 75 showed that the anatomic regions which most commonly suffered injuries with a severity of AIS3-5 were the lower extremities in 133 (81.6%), thorax in 130 (79.7%), abdominal and pelvic contents in 99 (60.7%), head in 95 (58.3%), and spine in 26 (15.9%).

### Injury Severity Score

There were no patients with an ISS less than 16 in the PFx group and this allows its definition as a severely injured group.

The median ISS of the PFx group was 50 (17-75) and the mean ISS 51.0 (SD ± 16.3), while the median ISS of the control group was 26 (1-75) and the mean ISS 30.6 (SD ± 18.9). The median ISS for the PFx group was significantly higher than the ISS for the control group (two-sample Wilcoxon rank-sum [Mann-Whitney] test, *p *< 0.0001).

### ISS and age

The PFx group and the control group were further categorised by ISS and age higher or lower than 55 years, as shown in Table 3 because age has a prognostic value. The PFx group included higher proportions in the ISS groups of 50-74 and 75, than the control group. Within the PFx group the ISS groups of 50-74 and 75 were more commonly found in the age group less than 55 years, while the less severe ISS groups were more common in the age group 55 and older.

**Table 3:** Categorisation of all 209 patients of the PFx group and all 761 patients of the control group by age and Injury Severity Score (ISS). All patients of the PFx group suffered severe trauma with an ISS equal to 17 or higher.

	Age < 55 years				Age ≥ 55 years			
	**PFx group**		**Control group**		**PFx group**		**Control group**	
	
**ISS-groups**	**N**	**%**	**N**	**%**	**N**	**%**	**N**	**%**

1-8*	0	0	3	1.1	0	0	21	4.3

9-15	0	0	21	7.9	0	0	91	18.4

16-24	3	3	40	15	4	3.7	103	20.9

25-40	20	19.8	100	37.5	27	25	188	38.1

41-49	14	13.9	35	13.1	22	20.4	30	6.1

50-74	40	39.6	29	10.9	33	30.6	34	6.9

75	24	23.8	39	14.6	22	20.4	27	5.5

Totals	101	100	267	100	108	100	494	100

*The 24 victims of the control group with ISS 1-8 suffered a major complication such as acute myocardial infarction, pulmonary embolism and multiple organ failure.

### Preventable deaths

Based on the definition of the study, 163 (78% of the total 209) deaths in the PFx group had an ISS less than 75 and were categorized as potentially preventable, while 46 (22%) had an ISS of 75 and were considered not preventable.

### Associated injuries

The complete range of recorded injuries was classified according to the AIS-90 anatomic regions and the AIS-90 severity codes, having first excluded the non-preventable deaths, i.e. 46 (22% of the total 209) from the PFx group and 66 (8.7% of the total 761) from the control group with an ISS equal to 75.

**Figure 1:**
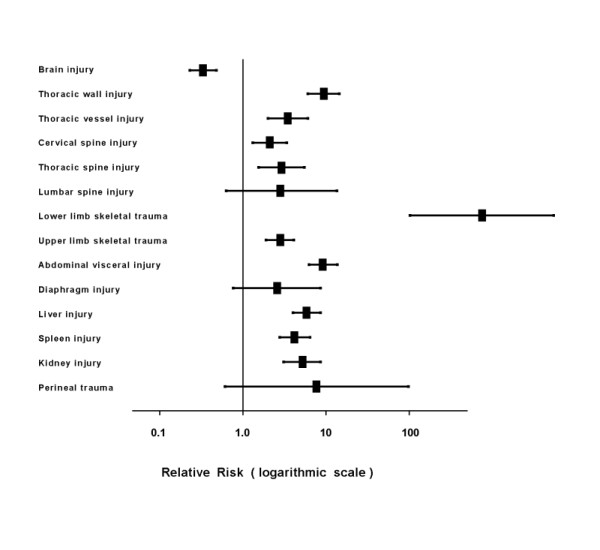
**Forest plot of injuries associated with pelvic fractures**. Odds ratios are adjusted for gender, age, intention, and height of fall, and are displayed on a logarithmic scale. The vertical line represents no association with pelvic fracture (odds ratio of 1.0). When the line for an injury does not cross the vertical line, there is a statistically significant association between the particular injury and pelvic fracture.

Hence, comparison of associated injuries was limited to a subset of 163 (78%) patients from the PFx group, versus a subset of 695 (91.3%) from the control group, all of whom had an ISS less than 75 and were classified as potentially preventable deaths. Univariate logistic regression analyses were performed and followed by multivariate logistic regression analysis to adjust for age, gender, intention, and height of fall. Multiplicity and severity of associated injuries were significantly more common in most anatomic regions in the PFx group than in the control group. The full results are shown in Table 4 and Figure 1.

**Table 4:** Comparison of associated injuries between the subsets of patients of the PFx group and the control group, who had an ISS of less than 75 and were classified as potentially preventable deaths. Associated severe injuries were significantly more common in the PFx group than in the control group.

**Injuries: Anatomic Region**, (AIS-90 codes)	PFx group		Control group		After adjustment for age, gender, intention, and height of fall		*p*-value
		
	n	%	n	%	OR†	95% CI	
Total number of patients analysed	163	100	695	100			

Head, (1.*.**.**.*), (AIS1-5)	112	68.7	575	82.7	0.42	0.28-0.63	< 0.001

Brain, (1.4.**.**.*), (AIS3-5)	92	56.4	535	77.0	0.33	0.23-0.49	< 0.001

Skull, (1.5.**.**.*), (AIS2-4)	47	28.8	393	56.6	0.27	0.18-0.40	< 0.001

Thorax, (4.*.**.**.*), (AIS1-5)	145	89.0	278	40.0	10.54	6.25-17.79	< 0.001

Thoracic wall, (4.5.**.**.*), (AIS2-5)	137	84.1	232	33.4	9.36	5.94-14.76	< 0.001

Thoracic viscera, (4.4.**.**.*), (AIS2-5)	99	60.7	129	18.6	5.90	4.01-8.66	< 0.001

Thoracic vessels (4.2.**.**.*), (AIS2-5)	28	17.2	36	5.2	3.47	2.00-6.02	< 0.001

Abdominal, (5.*.**.**.*), (AIS1-5)	117	71.8	145	20.9	8.36	5.54-12.59	< 0.001

Abdominal/pelvic viscera, (5.4.**.**.*), (AIS1-5)	112	68.7	123	17.7	9.07	6.03-13.66	< 0.001

Liver, (5.4.18.**.*), (AIS2-5)	76	46.6	78	11.2	5.83	3.86-8.79	< 0.001

Spleen, (5.4.42.**.*), (AIS2-5)	57	35.0	70	10.1	4.21	2.74-6.47	< 0.001

Gastrointestinal tract, (5.4.44.**.*, 5.4.10.**.*, 5.4.14.**.*, 5.4.08.**.*, 5.4.36.**.*), (AIS2-5)	3	1.8	8	1.2	1.16	0.29-4.56	0.834

Abdominal vessels, (5.2.**.**.*), (AIS2-5)	0	0.0	1	0.1	Cannot be estimated		

Diaphragm, (4.4.06.**.*), (AIS2-3)	5	3.1	6	0.9	2.57	0.75-8.83	0.133

Kidney, (5.4.16.**.*), (AIS2-5)	38	23.3	31	4.5	5.17	3.04-8.82	< 0.001

Bladder, (5.4.06.**.*), (AIS2-4)	0	0.0	1	0.1	Cannot be estimated		

Genitourinary tract, (5.4.48.**.*, 5.4.06.**.*, 5.4.50.**.*, 5.4.52.**.*, 5.4.34.**.*, 5.4.56.**.*, 5.4.54.**.*, 5.4.30.**.*, 5.4.46.**.*), (AIS1-5)	5	3.1	2	0.3	11.64	2.08-65.0	0.005

Perineum, (5.4.32.**.*), (AIS1-3)	2	1.2	1	0.1	7.77	0.60-100.0	0.116

Spine, (6.**.**.*.*), (AIS2-5)	45	27.6	98	14.1	2.40	1.57-3.65	< 0.001

Cervical spine, (6.3.02.**.*, 6.4.02.**.*, 6.5.02.**.*), (AIS2-5)	30	18.4	77	11.1	2.11	1.30-3.43	0.003

Thoracic spine, (6.3.04.**.*, 6.4.04.**.*, 6.5.04.**.*), (AIS2-5)	18	11.0	25	3.6	2.90	1.50-5.62	0.002

Lumbar spine, (6.3.06.**.*, 6.4.06.**.*, 6.5.06.**.*), (AIS2-5)	3	1.8	5	0.7	2.79	0.62-12.55	0.182

Upper extremities, (7.*.**.**.*), (AIS1-3)	102	62.6	279	40.1	2.07	1.44-2.99	< 0.001

Upper limb skeletal trauma, (7.5.**.**.*), (AIS1-3)	59	36.2	105	15.1	2.77	1.87-4.11	< 0.001

Humerus, (7.5.26.**.*), (AIS2-3)	29	17.8	37	5.3	3.37	1.96-5.81	< 0.001

Forearm, (7.5.28.**.*, 7.5.32.**.*), (AIS2-3)	29	17.8	23	3.3	5.79	3.17-10.57	< 0.001

Lower extremities, (8.*.**.**.*), (AIS1-5)	114	69.9	271	39.0	3.15	2.16-4.60	< 0.001

Lower limb skeletal trauma, (8.5.**.**.* besides pelvis: 8.5.26.**.*, 8.5.28.**.*, 8.5.30.**.*), (AIS1-3)	162	99.4	120	17.3	762.6	105.3-5525	< 0.001

Femur, (8.5.18.**.*), (AIS2-3)	45	27.6	74	10.7	3.20	2.06-4.97	< 0.001

Tibia, (8.5.34.**.*, 8.5.16.**.*), (AIS1-3)	38	23.3	38	5.5	4.44	2.68-7.36	< 0.001

Acetabulum-hip, (8.5.06.**.*, 8.5.26.00.2), (AIS1-2)	4	2.5	3	0.4	5.63	1.15-27.65	0.033

Foot fracture, (8.5.02.**.*, 8.5.04.**.*, 8.5.10.**.*, 8.5.12.**.*, 8.5.14.**.*, 8.5.20.**.*, 8.5.22.**.*, 8.5.32.**.*, 8.5.36.**.*), (AIS1-2)	8	4.9	8	1.2	3.48	1.24-9.79	0.018

Calcaneus fracture, (8.5.14.00.2), (AIS2)	3	1.8	1	0.1	11.80	1.14-121.9	0.038

*The asterisks that follow the AIS-90 codes indicate that the complete range of injuries was included. Injuries are ranked on a scale of 1 to 6, with 1 being minor, 2 moderate, 3 serious, 4 severe, 5 critical, and 6 not compatible with life injury.

§PFx group versus control group.

AIS-90, Abbreviated Injury Scale - 1990 revision; OR, odds ratio; PFx, pelvic fractures.

There were recorded data allowing classification of PFx as open or closed for 189 (90.4%) of the 209 patients of the PFx group. There were 3 (1.6% of 189) patients with open PFx and 186 (98.4% of 189) with closed PFx. The open PFx subset had a median ISS of 55 (41-75) and the closed PFx subset an ISS of 50 (17-75). In addition, the median post-traumatic survival time for the open PFx subset was 23 minutes (0-70 minutes) and for the closed PFx subset 30 minutes (0-1,180 hours and 45 minutes).

### Haemorrhage

Assessment of the subset of the PFx-group that consisted of 163 potentially preventable deaths (ISS less than 75), with respect to the presence of haemorrhage, revealed that 126 (60.3%) of the total 209 subjects with PFx, or 73.3% of the total 163 potentially preventable deaths of the PFx group, had at least one AIS-90 code listed in Table 1 which denoted a potential cause of major haemorrhage other than the PFx.

### Transfer time

Of the 209 patients in the PFx group, 116 (55.5%) were initially admitted to a hospital in the Greater Athens region and 6 (2.9%) to a hospital in the provinces; the remaining 87 (41.6%) were either not admitted to any hospital or their status was unknown.

Of the 761 patients of the control group, 500 (65.7%) were initially admitted to a hospital in the Greater Athens region and 70 (9.2%) to a hospital in the provinces; the remaining 191 (25.1%) were either not admitted to any hospital or their status was unknown.

The time elapsing from injury to arrival in the emergency department was estimated for the PFx group, having first excluded the 132 (63.2%) victims who were found dead at the scene and the 37 (17.7%) for whom data were missing. For the remaining 40 (19.1% of the total 209) patients, the median transfer time was 22.5 minutes (10-90 minutes).

The transfer time for the control group was estimated, having first excluded the 256 (33.6%) subjects who were found dead at the scene, 383 (50.3%) for whom the transfer time was missing and 2 (0.3%) subjects who were classified as secondary deaths. For the remaining 120 (15.8%) patients the median transfer time was 30 minutes (range 5 minutes to 30 hours).

There were no significant differences with respect to transfer time between the PFx and control groups.

### Comorbidities

The rates of reported, but non-stratified with respect to severity, comorbid conditions of the PFx group were compared to those of the control group after adjustment for gender, age, intention, and height of fall. There were no statistically significant differences between the two groups with respect to chronic obstructive airways disease, ischaemic heart disease, congestive heart failure, hypertension, diabetes mellitus, obesity, cirrhosis, or alcohol/drug dependency. However, an approximately 300% higher odds of having a psychiatric history was found in the PFx group compared to the control group (OR: 4.18, 95% CI: 2.52-6.95, *p *< 0.001).

### Surgical operations performed

Of the 209 patients in the PFx group, a subset of only 25 (11.9%) left the emergency department alive to undergo additional treatment. Nearly half of the patients needed a surgical operation and abdominal operations were significantly more common in the PFx than in the control group (see Additional file 3).

### Complications

Pneumonia was reported in two patients and pulmonary embolism in another two of the 25 who were admitted to a hospital.

### Deaths directly attributed to pelvic fractures

Applying all four predefined criteria limited the proportion of deaths directly attributed to pelvic fractures to six (2.9%), Table 5.

**Table 5:** Deaths directly attributed to pelvic fractures.

	N	%
PFx group.	209	100

Patients with PFx AIS ≤ 3 in severity who were excluded.	154	73.7

Remaining patients with PFx AIS4 and AIS5 in severity.	55	26.3

Remaining patients without any, other than PFx injury of AIS ≥ 4 in severity, in any other AIS anatomic region.	6	2.9

Post-injury survival time ≤ 48 h.	6	2.9

Patients without any injuries listed in Table 1, which denote a potential cause of major haemorrhage other than those related to PFx.	6	2.9

PFx: pelvic fracture; AIS: Abbreviated Injury Scale (the scale of injury severity ranges from 1 to 6, with 1 being minor, 2 moderate, 3 serious, 4 severe, 5 critical, and 6 an unsurvivable injury).

### Post-traumatic survival time

**Figure 2:**
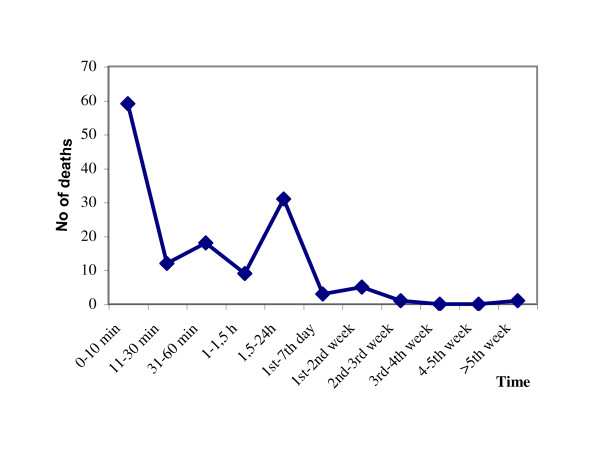
**Distribution of 139 of the 209 (66.5%) patients of the PFx group over the corresponding post-traumatic survival time substantiates the time limits during the early management**.

The exact time of injury or time of death was missing for 70 patients in the PFx group and for 221 patients in the control group. Comparison of the post-traumatic survival time of the remaining 139 (66.5%) patients of the PFx group, versus the post-traumatic survival time of the 540 (71.0%) patients of the control group revealed that the median post-traumatic survival time for the PFx group was 30 minutes and for the control group 20 hours and 15 minutes. The difference between the groups was highly significant (two-sample Wilcoxon rank-sum [Mann-Whitney] test, *p *< 0.001). The distribution of deaths over the corresponding post-traumatic survival time for the PFx-group is shown in Figure 2.

### Correlation of post-traumatic survival time with the ISS-groups

**Figure 3:**
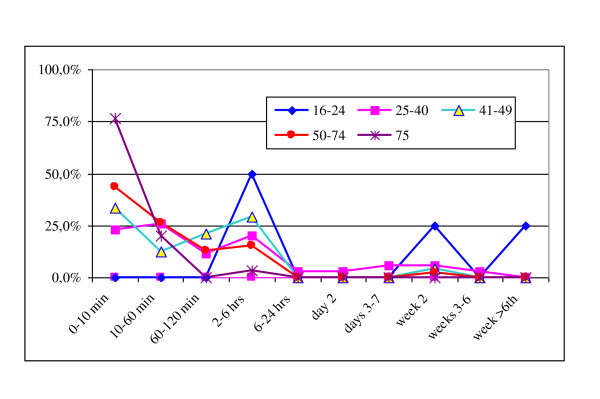
**Correlation of post-traumatic survival time with the ISS groups**. The post-traumatic survival time was known for 139 of the 209 (66.5%) patients of the PFx group. There were no subjects with an ISS of less than 17. The percentage of deaths in the ISS groups 16-24, 25-40, 41-49, 50-74, and 75, over the corresponding post-traumatic survival time confirm that the most severely injured patients die earlier than the less severely injured.

A subset of 139 (66.5%) patients of the PFx group for whom the posttraumatic survival time was known was classified with respect to severity in the following ISS-groups, 16-24, 25-40, 41-49, 50-74, and 75, and the percentages of deaths over the following post-traumatic survival time intervals less than 10 min, 10-60 min, 60-120 min, 2-6 hours, 6-24 hours, 1-3 days, 3-7 days, 1-3 weeks, 3-6 weeks, more than 6 weeks, were compared after adjustment for age, gender, height of fall, and intention for the injury. Ordered logistic regression analysis was used and revealed that for a one-group increment in the range of ISS groups, the probability of survival was reduced by 57% (OR: 0.43, 95%CI, 0.32-0.58, *p *< 0.0001), Figure 3.

### Location of deaths

With respect to the location of death, a significantly higher proportion of patients, i.e. 147 (70.3%) of the PFx group were found dead at the scene or were dead on arrival, versus the 325 (42.7%) patients of the control group (Pearson chi-squared [6 df] = 140.31, *p *< 0.001).

From the PFx group, a subset of 184 (88%) who died at the scene, were dead on arrival, or died in the emergency department had a median ISS of 50 (19-75), while the remaining 25 (12%) patients who left the emergency department alive for additional treatment had a median ISS of 38 (17-75). The difference was statistically significant (two-sample Wilcoxon rank-sum (Mann-Whitney) test, p < 0.001). The relevant data are shown in Table 6.

**Table 6:** Location of death of the 209 patients in the PFx group.

	PFx group			Control group		
**Location of death**	**n**	**%**	**Median ISS (range)**	**n**	**%**	**Median ISS (range)**

Found dead	132	63.2	57 (24-75)	256	33.6	41 (5-75)

Dead on arrival	15	7.1	38 (19-75)	69	9.1	19 (1-75)

Emergency department death	37	17.7	43 (22-75)	53	7	29 (3-75)

Operating room death	10	4.8	43 (22-75)	10	1.3	34.5 (10-54)

Intensive care unit death	11	5.3	34 (17-50)	126	16.6	26 (9-75)

Ward death	4	1.9	32 (17-50)	226	29.7	18 (1-59)

*Secondary death	0	0	0	21	2.8	9 (4-54)

Total	209	100	50 (17-75)	761	100	26 (1-75)

*Secondary deaths were defined as those that occurred after the discharge of the patient from the hospital. ISS: Injury Severity Score; PFx: pelvic fracture.

## Discussion

### Main results

Comparison of the PFx group with the control group documented that PFx is a paradigm of injury that selected a population from the total fatal falls that shared common characteristics. The relevance of the trauma outcome variables (template) used to compare the two groups and their contribution to audit is further discussed herein.

### Mortality due to PFx

Falls are the second most common cause of trauma-related deaths, after MVC in Greece and in North America [8,9]. The management of patients with PFx has been widely discussed elsewhere [10-13]. In two large-scale studies [7,14], the reported in hospital mortality of patients with PFx was 13.5% and 17.7% respectively. However, PFx are associated with higher mortality rates when pre hospital trauma fatalities are included in the studies.

### The need for systematic audit of trauma care

The need for an evidence-based transformation of the system to an optimal [15] trauma care system in Greece, has been recognised and substantiated by other studies [16-21]. Data such as those produced by the present method can contribute to essential components of a nationally-based registry [22,23].

### Sample size

The present study is representative as it included approximately 46% (n = 970) of the 2,102 deaths due to falls (ICD-9, E50), which occurred throughout Greece for the study period 1996 to 2000.

### Rates of PFx

Selection of subjects with PFx reduced the sample size to 21.5% (209) of the total 970 subjects who suffered a fatal fall. Hence, a reduced number of autopsies are required to repeat the audit. Importantly, similar proportions of patients with PFx (17.8% [24], 23% [25], 23% [26], (25.4%) [2] have been reported in trauma autopsy and clinical studies and indicate the availability of subjects on which to base auditing procedures.

### Risk factors for PFx

In the present study, multivariate analysis revealed that gender, age, intention for the accident, and height of fall increased the probability of suffering a PFx after a fall. Hence, the PFx group shared common risk factors. Materials structured by the described method allowed the identification of common risk factors for PFx which are important for developing primary prevention programmes [27].

### Gender

The present study has shown that men had 28% lower odds of developing a PFx following a fall compared to females. Hence, the PFx used as a paradigm of injury selected a lower proportion of men than women from the total sample. Data on the predictive value of gender on the trauma outcome are still controversial [28,29].

### Age

The present study revealed that age was a notable predictor because each one-year increment of age was associated with a decrease in the odds of suffering a PFx after a fall by 1.2%. This finding is skewed from that of a previous study [2] and reaffirms that different age-groups are prone to suffering trauma via different mechanisms.

Moreover, age is a well-known predictive factor following trauma [5] and is a vital parameter for audit. It has been reported that increased mortality starts at the age of 40 years [30], but other authors have reported that the cut-off of age-dependent mortality at the age of 56 years and this increase is independent of the injury severity [31]. Evidence-based guidelines with respect to management of elderly trauma patients have been proposed elsewhere [32].

### Location of injury, rural trauma and the need for regionalisation of care

In the present study, the observed significantly higher risk for a PFx after a fall in the Greater Athens region than in the provinces reflects differences with respect to professional and other activities of the patients, between the capital and the provinces.

Rural trauma is associated with a higher mortality rate than urban trauma [33]. Mapping the location of injury should indicate the environmental factors predisposing to trauma. Key issues for effective upgrading of the care of trauma patients in rural regions are comprehensively discussed elsewhere [34].

### Intention for the injury

In the present study, people who had the intention of committing suicide had a higher probability of developing a PFx after a fall compared to those who did not have such an intention. The reported suicide rate for Greece in 1997 was 2.8 per 100,000 citizens [35]. Suicide is a huge but preventable public health problem [36]. The data of the present study indicate that prevention measures should be re-addressed in Greece.

Unintentional falls at workplaces, homes, and those related to childhood and sporting activities are important concerns [37].

In this study, the height of fall was a notable predictor, because for a one-metre increment in the height of fall the rates of PFx increased (*p *< 0.001). SF in the elderly, are not so simple because the relative risk of in-hospital death is 15 times higher in the elderly compared with patients younger than 65 years [38]. Safety measures are fields for preventive actions [27,39,40].

### Mechanism of injury classified by the ICD-9

The mechanism of injury provides an insight into anticipated patterns of injury. Data classified according to ICD-9, as in the present study, allow global comparisons. In addition, derivatives of ICD-9 have been shown to outperform both ISS and TRISS as predictors of trauma survival, hospital charges and length of hospital stay [41].

### Toxicology

The presence of alcohol in 17.2% of the PFx group and in 19.3% of the control group was the most common pre-injury co-morbid condition. The emerging concept of preventive interventions by trauma centres with respect to alcohol and illicit drugs should be addressed in this country also [42].

### Severity assessment: AIS-90 anatomic regions and severity codes

Severity of injury is the most important determinant of survival following injury. In the present study, analysis of 163 (78% of the total 209) potentially preventable deaths (ISS less than 75) outlined the distribution and relative frequencies of the injuries in the anatomic regions as well as their AIS-90 severity codes. AIS-90 severity codes and anatomic regions were indispensable in classifying and comparing the groups of patients with PFx to those without.

### ISS

In the present study, the PFx used as a paradigm selected more severely injured patients than the control group and this has been substantiated as all subjects of the PFx group had an ISS equal to or higher than 17, the median ISS of the PFx group was 50 (17-75), while the median ISS of the control group was 26 (1-75) and the difference between the groups was statistically significant. Distribution of deaths in the PFx group in relation to the ISS showed that patients with a higher ISS died during earlier stages of medical care than those with a lower ISS. An ISS higher than 25 has been reported to be a risk factor associated with mortality of patients with PFx [7].

The postmortem-based ISS may differ from clinically estimated antemortem ISS [43] and earlier studies on preventable trauma deaths that were based on autopsy reports were strongly criticised as being inconsistent and subjective [44,45]. However, in the present study the autopsy reports were used to extract the description of the injuries that were consequently classified by the AIS-90 anatomic descriptions, severity codes, and ISS.

Omitting the pre-hospital trauma deaths from analyses would affect the spectrum of severity descriptions as well as the epidemiological data [22] and potentially the trauma care policies.

### Associated injuries

The present study has reaffirmed that the associated injuries coded according to AIS-90 in the potentially preventable (ISS less than 75) subset were a significantly more common feature in the PFx group than in the control group (Table 4 and Figure 1).

Falls from heights result in visceral, vascular, and skeletal injuries [10,24,26].

Large energy absorption is required to produce a PFx and the energy dissipation caused many associated injuries. The present study indicated that when selecting the PFx paradigm a reproducible population should be selected, thus allowing data comparison.

### Deaths directly attributed to PFx

Based on the definitions of the study, 2.9% (n = 6) of the deaths were attributed to PFx *per se*, while the remainder had multiple other associated injuries, which led to death. The reported overall in hospital mortality of subjects with PFx is 13.5%, but only 0.8% of the deaths were attributed to PFx [7].

### Control of haemorrhage is a primary issue for audit

AIS-90-coded injuries, shown in Table 1 that denoted a potential cause of major haemorrhage other than the PFx, were encountered in 126 (60.3%) of the 209 subjects with PFx in the present study. Haemodynamic stability is a predictor of mortality [46], as unstable patients with PFx have mortality rates of 40-50% [47-49]. Hence, the timeliness of interventions performed on the patients is inevitably a subject for audit. A protocol [50] and guidelines [32] for the initial management have been recommended elsewhere.

### Comorbidity

In the present study, age was an indication of the functional reserve capacities of the patients [51,52]. Comorbidity is a known trauma outcome predictor [30,53-55] and a key issue that is needed to compare groups of patients when auditing trauma care [56,57].

A number of non-stratified diagnoses were evaluated but their role has not been elucidated in the present study. For patients who arrive alive at hospital the physical status can be classified by the American Society of Anesthesiologists (ASA) score [58,59], the co-morbidity by the Charlson co-morbidity index [60,61] and the APACHI II pre-injury chronic pathology set of definitions [55,62]. However, for a more accurate autopsy-based audit there is a need to develop autopsy-based grading of the severity of these common co-morbid conditions.

### Triage

Physiology derangement, anatomic injury diagnosis, mechanism of injury, pre-existing comorbid conditions [57] and ASA [59] are commonly used to triage trauma patients. Trauma score alone does not seem adequate for pre-hospital triage of patients with PFx [63]. In the present study, the significantly higher ISS values as well as the shorter post-traumatic survival time of the PFx group in comparison to controls, substantiate the rational practice which gives priority in transferring subjects with suspicion of PFx to high-level trauma centres [11].

### Transfer time and the Emergency Medical Care Service

Despite the relatively short median transferring times, some patients were transferred to the first available hospital and others to more organised units capable of managing major trauma, a fact that raises the issue of regionalisation of care in this country [64,65]. The Greek national EMCS, named EKAB [66], is the major means of trauma patients' transportation. However, data to audit its effectiveness are not regularly published [67,68].

### Performed surgery

The patterns of injuries that occur in falls were previously reported [10,26] and are comprehensively analysed in the present study, Table 2 and 4.

The pathophysiology [69] as well as the management of patients are discussed elsewhere [10,70,71]. In the present study abdominal surgery was significantly more commonly performed on the PFx group than on the control group. The proportion of patients who required a timely surgical intervention in relation to those who actually received this management is of paramount importance to assess standards of care, but the data do not allow any conclusions.

### Complications

The rates and stratification of severity of the most common in hospital complications are essential outcome measures. Avoiding errors in management, assuring effective treatment of multiple organ failure, sepsis, and pulmonary embolism would decrease trauma mortality [72]. To allow wide comparisons clear definitions of complications should be followed [73].

### Correlation of post-traumatic survival time to ISS

The median post-traumatic survival time for the PFx group was 30 minutes, while for the control group it was 20 hours and 15 minutes. The difference between the groups was highly significant and substantiates the time limits during the initial management of patients with PFx.

The correlation of the post-traumatic survival rates with the corresponding times to death revealed that as the severity (assessed by the ISS) increases, the survival rate of subjects of the PFx group decreases, Figure 3. Correlation of the ISS groups with the corresponding post-traumatic survival time should be a basic indicator for comparisons and audit.

### Location of death in relation to ISS group

A higher proportion of patients in the PFx group died sooner than those in the control group and did not have the opportunity for in hospital medical care. This was substantiated in the present study, as a significantly higher proportion of patients, i.e. 70.4% (n = 147) of the PFx group were found dead at the scene or were dead on arrival at hospital, versus 42.7% (n = 325) of the patients of the control group, a fact that reflects the higher severity of injury in the PFx group.

Previously reported rates of trauma deaths in the pre-hospital phase were 60.3% [74], and for trauma occurring in rural areas 70.5% [33]. It is of primary importance for auditing trauma care to know the proportion of potentially preventable deaths (ISS less than 75) among the patients who died without receiving specialised in-hospital care. The location of death in relation to ISS group should be a major index of the standards of trauma care.

### Non-preventable deaths as an argument for primary prevention programmes

In the present study 46 (22% of the total 209) patients with PFx were classified as non-preventable deaths (ISS equal to 75). This subset of deaths can only be avoided by primary injury prevention programmes. More than half of all trauma deaths are potentially preventable with pre-injury behavioural changes. Injury prevention is critical for reducing deaths in modern trauma systems [72] and the present study produced evidence for the establishment of prevention programmes as discussed earlier. The ratio of potentially preventable (ISS less than 75) to non-preventable deaths (ISS equal to 75) was 3.5:1 (163:46). These data should be used for future comparisons.

### Consistency of the presented data with standards of reporting trauma data

With respect to the standards for uniform reporting of data following major trauma, the present study showed that a substantial number of variables previously recommended [75] can be incorporated in an autopsy-based audit study.

### What makes patients with PFx a paradigm?

Skewing of the PFx population to the control group with respect to epidemiological data was shown. However, the most striking differences between the PFx group and the control group were the different risk factors and mechanisms of injury, the multiplicity of the associated injuries, the severity of the associated injuries, the high rates of injuries that caused major haemorrhage and the short post-traumatic survival time, which were significantly more commonly encountered in the PFx group. The described population with PFx shared common characteristics, and can be identified by the described methodology, allowing their definition as a paradigm of population. The described method produced a comprehensive overview of current trauma care.

### Study limitations

The main limitations of this study are its retrospective design and the lack of survivors for comparisons. The lack of systematic pre-mortem physiology data precluded the application of functional scores such as the revised trauma score [76] and the TRISS methodology for audit [77]. Standardised autopsy reports as well as autopsy based grading of comorbidity and refinement of the definition of deaths attributable to PFx should increase the validity of the method.

### Is the described method of autopsy data analysis reproducible and sustainable?

In the present study, the method of autopsy data analysis produced similar results to those of a previous study [2] from the same institutions. Reproduction by other authors remains to be seen. The applicability of this template was confirmed and should ensure a selection of comparable trauma populations. However, adjusted models of autopsy reports for trauma victims [78] as well as a systematic critical analysis of autopsy findings [79] is required to compare groups of subjects more accurately.

## Conclusions

The trauma population with PFx has characteristics that allow its definition as a paradigm of injury. Selection of the paradigm reduces the sample of an autopsy-based audit that renders systematic autopsy-based audit easier to apply. The structured template included a set of trauma outcome variables which substantially contributed to the audit of functional, infrastructural, management and prevention issues of the trauma care system and produced evidence for future comparisons. Further formulation of the method is a challenge for autopsy based trauma research.

## Abbreviations and acronyms

AIS-90: Abbreviated Injury Scale - 1990 revision; EMCS: Emergency medical care service; PFx: Pelvic fracture; ISS: Injury Severity Score; ICD-9: International Statistical Classification of diseases, injuries and causes of death - 9th revision; MVC: Motor vehicle collisions; Non-SF: Non-simple falls; OR: Odds ratio; SF: Simple fall

## Competing interests

The authors declare that they have no competing interests.

## Authors' contributions

INP, NKK: Study conception and design. INP, NKK, CL: Acquisition of data. INP, SB, GK, KP, SC, OK: Analysis and interpretation. INP: Drafting, writing of manuscript. All authors have read and approved the final version of the manuscript.

## Supplementary Material

Additional file 1 **Comparison of age and ISS between PFx and control groups**. The median age and the median Injury Severity Score (ISS), of the patients who were injured by the two most common categories of intention for the accident. Click here for file

Additional file 2 **Comparison of age and ISS between simple falls and non-simple falls**. Median age and Injury Severity Score (ISS) of the victims of simple falls (SF) and non-simple falls (non-SF). Click here for file

Additional file 3 **Performed Surgical Operations**. Performed Surgical Operations. Click here for file
