# Supplementary material for: Patients with pelvic fractures due to falls: A paradigm that contributed to autopsy-based audit of trauma in Greece
Source: J Trauma Manag Outcomes. 2011 Jan 8;5:2. doi: 10.1186/1752-2897-5-2 (PMC3024215; doi:10.1186/1752-2897-5-2)
Supplement: Additional file 1 — Comparison of age and ISS between PFx and control groups. The median age and the median Injury Severity Score (ISS), of the patients who were injured by the two most common categories of intention for the accident. [file 1752-2897-5-2-S1.DOC]

|  | PFx group | Control group | *PFx group versus control group,  p-values |
| --- | --- | --- | --- |
| Self-inflicted falls: Median age (range), in years | 54.5  (16–91) | 44  (15–90) | 0.006 |
| Unintentional falls: Median age (range), in years | 59.5  (20–96) | 70  (1–99) | 0.006 |
| Median age: Self-inflicted falls versus unintentional falls: p value | 0.188 | <0.001 |  |
| Self-inflicted falls: Median ISS (range) | 50  (17–75) | 42  (14–75) | 0.068 |
| Unintentional falls: Median ISS (range) | 44  (17–75) | 25  (1–75) | < 0.001 |
| Median ISS: Self-inflicted falls versus unintentional falls: p value | 0.121 | < 0.001 |  |

*Two-sample Wilcoxon rank-sum (Mann–Whitney) tests.
