# Supplementary material for: Patients with pelvic fractures due to falls: A paradigm that contributed to autopsy-based audit of trauma in Greece
Source: J Trauma Manag Outcomes. 2011 Jan 8;5:2. doi: 10.1186/1752-2897-5-2 (PMC3024215; doi:10.1186/1752-2897-5-2)
Supplement: Additional file 2 — Comparison of age and ISS between simple falls and non-simple falls. Median age and Injury Severity Score (ISS) of the victims of simple falls (SF) and non-simple falls (non-SF). [file 1752-2897-5-2-S2.DOC]

|  | PFx group | Control group | *PFx group versus control group, p-values |
| --- | --- | --- | --- |
| Simple falls: Median age (range), in years | 62  (22–96) | 73  (1–99) | p = 0.004 |
| Non-simple falls: Median age (range), in years | 54  (15–92) | 51  (1–94) | p = 0.49 |
| Simple falls: Median ISS (range) | 50  (22–75) | 21  (1–75) | p < 0.001 |
| Non-simple falls: (Median ISS, range) | 50  (17–75) | 38  (5–75) | p < 0.001 |

*Two-sample Wilcoxon rank-sum (Mann–Whitney) test, p < 0.001.
