# Supplementary material for: Patients with pelvic fractures due to falls: A paradigm that contributed to autopsy-based audit of trauma in Greece
Source: J Trauma Manag Outcomes. 2011 Jan 8;5:2. doi: 10.1186/1752-2897-5-2 (PMC3024215; doi:10.1186/1752-2897-5-2)
Supplement: Additional file 3 — Performed Surgical Operations. Performed Surgical Operations. [file 1752-2897-5-2-S3.DOC]

| Patients who left the emergency department alive for additional treatment | *PFx group | | Control group | | p-value** |
| --- | --- | --- | --- | --- | --- |
|  | n | % | n | % |  |
| All operating room, ICU, ward and secondary deaths | 25 | 100 | 383 | 100 |  |
| Any surgical operation | 13 | 52 | 151 | 39.4 | 0.214 |
| Abdominal surgery | 10 | 40 | 28 | 7.3 | < 0.001 |
| Orthopaedic surgery | 1 | 4 | 19 | 5 | 0.83 |
| Neurosurgery | 2 | 8 | 110 | 28.7 | 0.024 |
| Thoracic operation | 0 | 0 | 4 | 1 |  |

*PFx: Pelvic fracture. **Pearson Chi-squared tests.
